# Measures of Physical Performance and Muscle Strength as Predictors of Fracture Risk Independent of FRAX, Falls, and aBMD: A Meta‐Analysis of the Osteoporotic Fractures in Men (MrOS) Study

**DOI:** 10.1002/jbmr.3556

**Published:** 2018-08-29

**Authors:** Nicholas C Harvey, Anders Odén, Eric Orwoll, Jodi Lapidus, Timothy Kwok, Magnus K Karlsson, Björn E Rosengren, Eva Ribom, Cyrus Cooper, Peggy M Cawthon, John A Kanis, Claes Ohlsson, Dan Mellström, Helena Johansson, Eugene McCloskey

**Affiliations:** ^1^ Medical Research Council (MRC) Lifecourse Epidemiology Unit University of Southampton Southampton UK; ^2^ National Institute for Health Research (NIHR) Southampton Biomedical Research Centre, University of Southampton and University Hospital Southampton NHS Foundation Trust Southampton UK; ^3^ Centre for Bone and Arthritis Research (CBAR) Sahlgrenska Academy University of Gothenburg Gothenburg Sweden; ^4^ Centre for Metabolic Bone Diseases University of Sheffield Sheffield UK; ^5^ Oregon Health & Science University Portland OR USA; ^6^ Department of Public Health and Preventive Medicine Division of Biostatistics Oregon Health and Science University Portland OR USA; ^7^ Department of Medicine & Therapeutics and School of Public Health The Chinese University of Hong Kong Hong Kong The People's Republic of China; ^8^ Clinical and Molecular Osteoporosis Research Unit Lund University, Lund, Sweden; and Department of Orthopedics Skane University Hospital Malmö Sweden; ^9^ Department of Surgical Sciences University of Uppsala Uppsala Sweden; ^10^ National Institute for Health Research (NIHR) Biomedical Research Centre University of Oxford Oxford UK; ^11^ Research Institute California Pacific Medical Center San Francisco CA USA; ^12^ Department of Epidemiology and Biostatistics University of California–San Francisco San Francisco CA USA; ^13^ Institute for Health and Aging Catholic University of Australia Melbourne Australia; ^14^ Centre for Integrated Research in Musculoskeletal Ageing (CIMA) Mellanby Centre for Bone Research University of Sheffield Sheffield UK

**Keywords:** OSTEOPOROSIS, EPIDEMIOLOGY, FRAX, FALLS, FRACTURE, INTERACTION

## Abstract

Measures of muscle mass, strength, and function predict risk of incident fractures, but it is not known whether this risk information is additive to that from FRAX (fracture risk assessment tool) probability. In the Osteoporotic Fractures in Men (MrOS) Study cohorts (Sweden, Hong Kong, United States), we investigated whether measures of physical performance/appendicular lean mass (ALM) by DXA predicted incident fractures in older men, independently of FRAX probability. Baseline information included falls history, clinical risk factors for falls and fractures, femoral neck aBMD, and calculated FRAX probabilities. An extension of Poisson regression was used to investigate the relationship between time for five chair stands, walking speed over a 6 m distance, grip strength, ALM adjusted for body size (ALM/height^2^), FRAX probability (major osteoporotic fracture [MOF]) with or without femoral neck aBMD, available in a subset of *n* = 7531), and incident MOF (hip, clinical vertebral, wrist, or proximal humerus). Associations were adjusted for age and time since baseline, and are reported as hazard ratios (HRs) for first incident fracture per SD increment in predictor using meta‐analysis. 5660 men in the United States (mean age 73.5 years), 2764 men in Sweden (75.4 years), and 1987 men in Hong Kong (72.4 years) were studied. Mean follow‐up time was 8.7 to 10.9 years. Greater time for five chair stands was associated with greater risk of MOF (HR 1.26; 95% CI, 1.19 to 1.34), whereas greater walking speed (HR 0.85; 95% CI, 0.79 to 0.90), grip strength (HR 0.77; 95% CI, 0.72 to 0.82), and ALM/height^2^ (HR 0.85; 95% CI, 0.80 to 0.90) were associated with lower risk of incident MOF. Associations remained largely similar after adjustment for FRAX, but associations between ALM/height^2^ and MOF were weakened (HR 0.92; 95% CI, 0.85 to 0.99). Inclusion of femoral neck aBMD markedly attenuated the association between ALM/height^2^ and MOF (HR 1.02; 95% CI, 0.96 to 1.10). Measures of physical performance predicted incident fractures independently of FRAX probability. Whilst the predictive value of ALM/height^2^ was substantially reduced by inclusion of aBMD requires further study, these findings support the consideration of physical performance in fracture risk assessment. © 2018 The Authors. *Journal of Bone and Mineral Research* Published by Wiley Periodicals Inc.

## Introduction

The place of falls as a major risk factor for fracture is well established; the majority of hip fractures occur as a result of a fall from standing height or less.^1^, ^2^ There is also substantial evidence that risk factors related specifically to falls risk, such as physical performance, function, and muscle indices, are also related to the risk of incident fracture.^3^, ^4^, ^5^ Current clinical approaches to risk assessment are increasingly based on clinical risk factors, with or without aBMD, through fracture risk calculators. FRAX (fracture risk assessment tool) is the most commonly used fracture risk assessment tool worldwide,^6^ but unlike other tools such as QFracture or the GARVAN calculator,^7^, ^8^, ^9^ it does not include falls as a specific input risk factor^2^, ^10^ because of the inconsistent data across the 12 derivation and 11 validation cohorts.^11^ We have previously demonstrated that prior falls predict the risk of incident falls^12^ and fractures^13^ independently of FRAX probability. Although the predictive value of falls‐related risk factors for incident fracture have been demonstrated individually,^4^, ^5^ it has not been established whether the risk information so provided will be independent of that obtained through FRAX and aBMD. This is an important consideration because if these measures were to provide no additional information beyond the current fracture risk assessment, then there would be little to be gained from their measurement as part of fracture‐risk stratification. Furthermore, it is not clear whether specific falls risk factors, such as physical performance, might give information independent of the reporting of prior falls themselves. We therefore undertook a meta‐analysis of the three Osteoporotic Fractures in Men (MrOS) cohorts (United States, Sweden, Hong Kong) to investigate whether the predictive value of four measures (time for five chair stands, walking speed over a distance of 6 m, grip strength, and appendicular lean mass [ALM]) for incident fracture was independent of FRAX probability, history of falls, or aBMD.

## Subjects and Methods

### Participants

Details of the MrOS cohort studies have been published previously.^12^, ^13^, ^14^, ^15^ Briefly, MrOS is a multicenter study of community‐dwelling men aged 65 years or older from three countries, recruited and evaluated using similar criteria. To be eligible for the study, subjects had to be able to walk without aid. In the MrOS Hong Kong study, 2000 Chinese men, aged 65 to 92 years, were enrolled between August 2001 and February 2003.^16^ All were Hong Kong residents of Asian ethnicity. Stratified sampling was adopted to ensure that 33% of subjects were included in each of the following age groups: 65 to 69, 70 to 74, and ≥75 years. Recruitment notices were placed in housing estates and community centers for the elderly. In the MrOS Sweden study, 3014 men, aged 69 to 81 years, were enrolled between October 2001 and December 2004.^12^, ^17^ The cohort comprised men from the cities of Malmö, Gothenburg, and Uppsala, identified and recruited using national population registers. More than 99% were of Caucasian ethnicity. The participation rate in the MrOs Sweden study was 45%. In the MrOS United States study, 5994 men, aged 65 to 100 years, were enrolled at six sites between March 2000 and April 2002.^18^, ^19^ Each US clinical site designed and customized strategies to enhance recruitment of its population. Common strategies included mailings from the Department of Motor Vehicles, voter registration and participant databases, common seniors’ newspaper features and advertisements, and targeted presentations. Self‐defined racial/ethnic ancestry was ascertained through questionnaires at baseline.

### Exposure variables

At baseline, height (cm) and weight (kg) were measured, and BMI was calculated as kg/m^2^. The international MrOS questionnaire^18^ was administered at baseline to collect information about current smoking habits, number and type of medications, fracture history, family history of hip fracture, past medical history (rheumatoid arthritis), and high consumption of alcohol (three or more glasses of alcohol‐containing drinks per day), calculated from the reported frequency and amount of alcohol use. Previous fracture at baseline was documented as all fractures after the age of 50 years regardless of trauma. Glucocorticoid exposure was documented in MrOS as use at least 3 times per week in the month preceding the baseline assessment. Apart from glucocorticoid use and rheumatoid arthritis (both FRAX input variables), there was no information on secondary causes of osteoporosis and the “Secondary Osteoporosis” input variable for FRAX probability calculation was set to “No” for all men. Self‐reported falls during the 12 months preceding the baseline were recorded by questionnaire (past falls). Time for five chair stands, walking speed over 6 m (at usual pace), and grip strength using JAMAR dynamometers (Sammons Preston Rolyan, Bolingbrook, IL, USA) were assessed at the baseline visit. Areal bone mineral density (aBMD) was measured at the femoral neck and ALM from whole body scans using Hologic QDR 4500 A or W (Hologic, Bedford, MA, USA) or Lunar Prodigy (GE Lunar Corp., Madison, WI, USA) depending on the center, with cross calibration of instruments for aBMD. A *T*‐score was calculated using NHANES (National Health and Nutrition Examination Survey) young women as a reference value.^20^, ^21^ A 10‐year probability of fracture (FRAX major osteoporotic fracture: hip, humerus, vertebral, or forearm sites) was calculated using the clinical risk factors described above, with and without femoral neck aBMD entered into country‐specific FRAX models.

### Fracture and death outcomes

Hong Kong:^22^ Incident fractures were captured via subject follow‐up through a phone call or a visit to the research center. All fracture sites (hip, wrist, skull/face, ribs, shoulder, arm, wrist, vertebra, tibia, fibula, foot, metatarsal toes, hand, fingers, and pelvis) were recorded. Pathological fractures were excluded. All incident fractures reported by participants were then confirmed by X‐rays or medical records. Deaths were verified by death certificates.

Sweden:^23^ Central registers covering all Swedish citizens were used to identify the subjects and the time of death for all subjects who died during the study; these analyses were performed after the time of fracture validation. At the time of fracture evaluation, the computerized X‐ray archives in Malmö, Gothenburg, and Uppsala were searched for new fractures occurring after the baseline visit using the unique personal registration number allocated to every Swedish citizen. All additional fractures reported by the study subject after the baseline visit were confirmed by physician review of radiology reports. Fractures reported by the study subject, but not confirmed by radiographic report, were not included.

United States:^18^ If a participant reported a fracture, study staff conducted a follow‐up telephone interview to determine the date and time the fracture had occurred, a description of how the fracture occurred, the type of trauma that resulted in the fracture, the participant's location and activities at the time of the fracture, symptoms just before or coincident with the fracture, and source of medical care for the fracture. All reported fractures were centrally verified by a physician adjudicator through medical records obtained from the participant's physician. Deaths were verified through state death certificates.

### Statistical methods

Clinical outcomes comprised any fracture, osteoporotic fracture (defined according to Kanis et al., 2001^24^ as clinical vertebral, ribs, pelvis, humerus, clavicle, scapula, sternum, hip, other femoral fractures, tibia, fibula, distal forearm/wrist), major osteoporotic fracture (MOF: hip, clinical vertebral, humerus, or wrist/ forearm), and hip fracture. An extension of Poisson regression models^25^ was used to study the association between predictors, FRAX, prior falls, aBMD, and the future risk of fracture. All associations were adjusted for age and time since baseline. In contrast to logistic regression, the Poisson regression uses the length of each individual's follow‐up period and the hazard function is assumed to be exp(β_0_ + β_1_ – current time from baseline + β_2_ – current age + β_3_ – variable of interest). The observation period of each participant was divided into intervals of one month. One fracture per person and time to the first fracture were counted; events were censored if they occurred after the time of first fracture, loss to follow‐up, death, or end of follow‐up. To correct for body size, ALM for each individual was divided by the square of their height. We initially investigated the predictive value of each of the four exposures (chair stand time, walking speed, grip strength, and ALM/height^2^, all standardized to be normally distributed with mean = 0 and SD = 1) adjusted only for age and follow‐up time. Subsequently, we used multivariate models to investigate the predictive value of these indices independent of FRAX, prior falls, or aBMD (entered into the model as femoral neck *T*‐score). Additionally, we investigated whether inclusion of BMI or levels of physical activity (Physical Activity Scale for the Elderly [PASE] questionnaire^26^) modified the associations, and also explored the predictive value of ALM/BMI. In further analyses, we investigated interactions with age and time since baseline, in which age and time were used as continuous variables and examples given at specific ages and times. The association between predictive factors and risk of fracture are described as a hazard ratio (HR) per 1 SD change in predictor together with 95% confidence intervals (CIs). Two‐sided *p*‐values were used for all analyses; *p* < 0.05 was considered to be significant. Analyses were undertaken separately within each cohort; then the β‐coefficients from each cohort were weighted according to the variance and merged to determine the weighted mean of the coefficient and its SD (fixed‐effects meta‐analysis because heterogeneity was low to moderate as assessed by I^2^).^27^ The risk ratios are then given by e^(weighted mean coefficient)^.

## Results

### Characteristics of participants

The study cohort consisted of 10,411 men who had information on the key exposures, together with prior falls and femoral neck aBMD: 5660 men in the United States (mean age 73.5 years; mean follow‐up 10.9 years), 2764 men in Sweden (mean age 75.4 years; mean follow‐up 8.7 years), and 1987 men in Hong Kong (mean age 72.4 years; mean follow‐up 9.9 years). The frequency of past falls was similar across the cohorts at 20%, 16%, and 15%, respectively. Previous fractures were more commonly reported in Sweden (35%) than in the United States (22%) and Hong Kong (14%). Consistent with the known country‐specific epidemiology of fracture, the highest mean FRAX probability (major osteoporotic fracture [MOF] with aBMD) was observed in Sweden (11.4%), followed by the United States (7.8%) and Hong Kong (6.6%). There were 61 men (0.6%) who were unable to complete the chair stand test. Summary statistics for the key exposure variables are presented in Table 1, which summarizes the baseline characteristics of the individuals by country cohort.

**Table 1:** Baseline Characteristics and Fracture Outcomes of Study Participants by Country

	Hong Kong	Sweden	USA
Proportion of whole cohort	99%	92%	94%
*n*	1987	2764	5660
Person‐years	19,592	24,102	61,456
Age [mean (range)], years	72.4 (65–92)	75.4 (70–81)	73.5 (64–100)
BMI	23.5 ± 3.1	26.3 ± 3.5	27.4 ± 3.8
Previous fracture	14%	35%	22%
Family history hip fracture	5%	13%	17%
Smoker	12%	8%	3%
Glucocorticoids	1%	2%	2%
Rheumatoid arthritis	1%	1%	5%
Excess alcohol	1%	2%	4%
aBMD FN T‐score	−1.4 ± 0.9	−0.9 ± 1.0	−0.6 ± 1.1
Time 5 stands (s)	12.7 ± 3.9	13.4 ± 4.2	11.1 ± 3.3
Walk speed (m/s)	1.0 ± 0.2	1.3 ± 0.3	1.2 ± 0.2
Fall	15%	16%	20%
Grip strength (kg)	33.9 ± 6.7	43.1 ± 7.8	41.8 ± 8.4
ALM (kg)	20.2 ± 2.8	24.3 ± 3.2	24.3 ± 3.5
Height (cm)	163 ± 5.7	175 ± 6.5	174 ± 6.8
ALM/height^2^	7.6 ± 0.9	7.9 ± 0.8	8.0 ± 0.9
FRAX MOF without aBMD	6.9 ± 2.9	13.5 ± 6.1	9.1 ± 4.8
FRAX hip without aBMD	3.4 ± 2.5	7.5 ± 5.5	3.6 ± 3.9
FRAX MOF with aBMD	6.6 ± 3.2	11.4 ± 6.7	7.8 ± 4.5
FRAX hip with aBMD	3.0 ± 2.6 (*n* = 1661)	5.5 ± 6.0 (*n* = 1732)	2.4 ± 3.4 (*n* = 4138)
FU (hip fx: mean (SD), years	9.9 (2.8)	8.7 (2.9)	10.9 (3.8)
Any fx	11%	22%	19%
Osteoporotic fx	9%	19%	15%
MOF fx	7%	16%	10%
OWH fx (MOF)	4%	12%	5%
Hip fx	3%	7%	4%

FN = femoral neck; ALM = appendicular lean mass; FU = follow‐up; FRAX = fracture risk assessment tool; fx = fracture; MOF = major osteoporotic fracture; OWH = osteoporotic fracture without hip fracture.

### Associations between chair stand time, walking speed, grip strength, appendicular lean mass, and incident fracture risk

Table 2 summarizes the associations between each of the four predictors (chair stand time, walking speed, grip strength, and ALM divided by height^2^, adjusted only for age and follow‐up time), and the fracture outcomes. Thus, across all cohorts, greater time for five chair stands was associated with a greater risk of MOF (HR 1.26; 95% CI, 1.19 to 1.34), whereas greater walking speed (HR 0.85; 95% CI, 0.79 to 0.90), grip strength (HR 0.77; 95% CI, 0.72 to 0.82) and ALM/height^2^ (HR 0.85; 95%CI, 0.80 to 0.90) were associated with a lower risk of incident MOF. Results for any fracture, osteoporotic fracture, and hip fracture outcomes were very similar, as were associations by cohort.

**Table 2:** Associations Between Exposures and Risk of Incident Fracture

		Any fx	Ost fx	MOF fx	Hip fx
Time 5 chair stands	HK	1.13 (0.99, 1.30)	**1.19 (1.02, 1.38)**	**1.24 (1.04, 1.46)**	1.20 (0.93, 1.55)
	SW	**1.14 (1.06, 1.24)**	**1.21 (1.11, 1.31)**	**1.21 (1.10, 1.33)**	**1.38 (1.19, 1.60)**
	US	**1.17 (1.10, 1.24)**	**1.18 (1.10, 1.26)**	**1.30 (1.20, 1.42)**	**1.38 (1.21, 1.58)**
	**Total**	**1.15 (1.10, 1.21)**	**1.19 (1.13, 1.25)**	**1.26 (1.19, 1.34)**	**1.36 (1.24, 1.49)**
Walking speed	HK	**0.84 (0.73, 0.97)**	**0.80 (0.68, 0.94)**	**0.78 (0.65, 0.93)**	**0.57 (0.44, 0.75)**
	SW	**0.86 (0.79, 0.93)**	**0.84 (0.77, 0.91)**	**0.84 (0.76, 0.92)**	**0.72 (0.62, 0.84)**
	US	0.95 (0.89, 1.02)	**0.93 (0.86, 1.00)**	**0.87 (0.79, 0.95)**	**0.73 (0.63, 0.84)**
	**Total**	**0.91 (0.86, 0.95)**	**0.88 (0.83, 0.93)**	**0.85 (0.79, 0.90)**	**0.70 (0.64, 0.77)**
Grip strength	HK	**0.76 (0.66, 0.88)**	**0.77 (0.66, 0.91)**	**0.75 (0.63, 0.90)**	**0.71 (0.54, 0.93)**
	SW	**0.79 (0.73, 0.86)**	**0.78 (0.71, 0.85)**	**0.76 (0.69, 0.84)**	**0.69 (0.59, 0.80)**
	US	**0.86 (0.81, 0.92)**	**0.80 (0.74, 0.86)**	**0.78 (0.71, 0.86)**	**0.74 (0.64, 0.86)**
	**Total**	**0.83 (0.79, 0.86)**	**0.79 (0.75, 0.83)**	**0.77 (0.72, 0.82)**	**0.72 (0.65, 0.79)**
ALM/Height^2^	HK	0.88 (0.76, 1.01)	**0.84 (0.72, 0.99)**	**0.82 (0.69, 0.98)**	**0.74 (0.56, 0.97)**
	SW	**0.85 (0.78, 0.92)**	**0.84 (0.76, 0.91)**	**0.82 (0.75, 0.91)**	**0.84 (0.72, 0.98)**
	US	**0.91 (0.85, 0.96)**	**0.92 (0.85, 0.99)**	**0.89 (0.81, 0.97)**	0.91 (0.79, 1.04)
	**Total**	**0.89 (0.84, 0.93)**	**0.88 (0.83, 0.93)**	**0.85 (0.80, 0.90)**	**0.86 (0.78, 0.95)**

Data are hazard ratios (HRs) for fracture (fx) per 1 SD increase in predictor (HR/SD), adjusted for age and follow‐up time. Statistically significant associations (*p* < 0.05) are in bold.

HK = Hong Kong; SW = Sweden; US = United States; fx = fracture; Ost = osteoporotic; MOF = major osteoporotic fracture.

### Independent predictive value of exposures after adjustment for prior falls or FRAX probability

The results of models additionally including prior fall or FRAX (MOF with or without aBMD) are documented in Table 3. The associations between each of the four exposures and any of the fracture outcomes remained very similar with adjustment for prior falls. The inclusion of FRAX [MOF without aBMD (using the subset of 7531 for whom FRAX probability could be calculated)] very slightly attenuated the magnitude of the HRs; in contrast, although inclusion of FRAX (MOF with aBMD) led to a modest attenuation of HRs in general, those for any fracture (HR 0.95; 95% CI, 0.90 to 1.01) and osteoporotic fracture (HR 0.95; 95% CI, 0.89 to 1.01) with ALM/height^2^ became nonsignificant, and that between ALM/height^2^ and MOF was also attenuated (HR 0.92; 95% CI, 0.85 to 0.99). Adjustment for BMI or physical activity also did not materially alter the magnitude of the relationships and associations for ALM were similar to those for ALM/height^2^. However, with ALM/BMI as the exposure, the patterns were again of similar direction, but were attenuated such that none of the models achieved statistical significance (summarized in Supplementary Table 1).

**Table 3:** Associations Between Exposures and Risk of Incident Fracture

Exposure (SD)	Adjustment	Any fx	Ost fx	MOF fx	Hip fx
Time 5 chair stands	Age, FU time	**1.15 (1.10, 1.21)**	**1.19 (1.13, 1.25)**	**1.26 (1.19, 1.34)**	**1.36 (1.24, 1.49)**
	+ prior falls	**1.15 (1.09, 1.20)**	**1.18 (1.12, 1.24)**	**1.24 (1.17, 1.31)**	**1.34 (1.23, 1.47)**
	or + FRAX wo aBMD	**1.13 (1.07, 1.20)**	**1.17 (1.10, 1.24)**	**1.26 (1.17, 1.35)**	**1.31 (1.17, 1.46)**
	or + FRAX with aBMD	**1.12 (1.06, 1.19)**	**1.16 (1.09, 1.23)**	**1.24 (1.15, 1.34)**	**1.29 (1.15, 1.44)**
	or + FN aBMD	**1.16 (1.11, 1.21)**	**1.19 (1.13, 1.25)**	**1.26 (1.19, 1.34)**	**1.35 (1.23, 1.48)**
Walking speed	Age, FU time	**0.91 (0.86, 0.95)**	**0.88 (0.83, 0.93)**	**0.85 (0.79, 0.90)**	**0.70 (0.64, 0.77)**
	+ prior falls	**0.91 (0.87, 0.95)**	**0.88 (0.83, 0.93)**	**0.85 (0.80, 0.90)**	**0.71 (0.65, 0.79)**
	or + FRAX wo aBMD	**0.88 (0.83, 0.93)**	**0.85 (0.80, 0.91)**	**0.82 (0.76, 0.88)**	**0.70 (0.62, 0.78)**
	or + FRAX with aBMD	**0.89 (0.84, 0.95)**	**0.85 (0.80, 0.91)**	**0.83 (0.77, 0.90)**	**0.71 (0.63, 0.80)**
	or + FN aBMD	**0.90 (0.86, 0.94)**	**0.87 (0.83, 0.92)**	**0.84 (0.79, 0.89)**	**0.71 (0.65, 0.78)**
Grip strength	Age, FU time	**0.83 (0.79, 0.86)**	**0.79 (0.75, 0.83)**	**0.77 (0.72, 0.82)**	**0.72 (0.65, 0.79)**
	+ prior falls	**0.83 (0.79, 0.88)**	**0.79 (0.75, 0.84)**	**0.78 (0.73, 0.83)**	**0.72 (0.65, 0.80)**
	or + FRAX wo aBMD	**0.84 (0.79, 0.89)**	**0.81 (0.76, 0.87)**	**0.79 (0.73, 0.85)**	**0.74 (0.66, 0.84)**
	or + FRAX with aBMD	**0.85 (0.80, 0.90)**	**0.83 (0.77, 0.89)**	**0.81 (0.75, 0.87)**	**0.76 (0.68, 0.86)**
	or + FN aBMD	**0.86 (0.82, 0.90)**	**0.83 (0.78, 0.88)**	**0.82 (0.77, 0.87)**	**0.79 (0.71, 0.87)**
ALM/Height^2^	Age, FU time	**0.89 (0.84, 0.93)**	**0.88 (0.83, 0.93)**	**0.85 (0.80, 0.90)**	**0.86 (0.78, 0.95)**
	+ prior falls	**0.88 (0.84, 0.93)**	**0.88 (0.83, 0.93)**	**0.86 (0.80, 0.91)**	**0.86 (0.78, 0.95)**
	or + FRAX wo aBMD	**0.93 (0.88, 0.99)**	**0.93 (0.87, 0.99)**	**0.89 (0.82, 0.96)**	0.91 (0.81, 1.02)
	or + FRAX with aBMD	0.95 (0.90, 1.01)	0.95 (0.89, 1.01)	**0.92 (0.85, 0.99)**	0.95 (0.85, 1.07)
	or + FN aBMD	1.01 (0.96, 1.06)	1.02 (0.96, 1.08)	1.02 (0.96, 1.10)	**1.12 (1.01, 1.23)**

Data are hazard ratios (HRs) for fracture (fx) per 1 SD change in predictor (HR/SD), adjusted for age, follow‐up time, and additional adjustment for either prior falls, FRAX MOF without femoral neck aBMD, FRAX MOF with femoral neck aBMD, femoral neck aBMD. Statistically significant associations (*p* < 0.05) are in bold.

fx = fracture; Ost = osteoporotic; MOF = major osteoporotic fracture; FU = follow‐up; FRAX = fracture risk assessment tool; FN = femoral neck.

### Independent predictive value of exposures after adjustment for femoral neck aBMD

**Figure 1:**
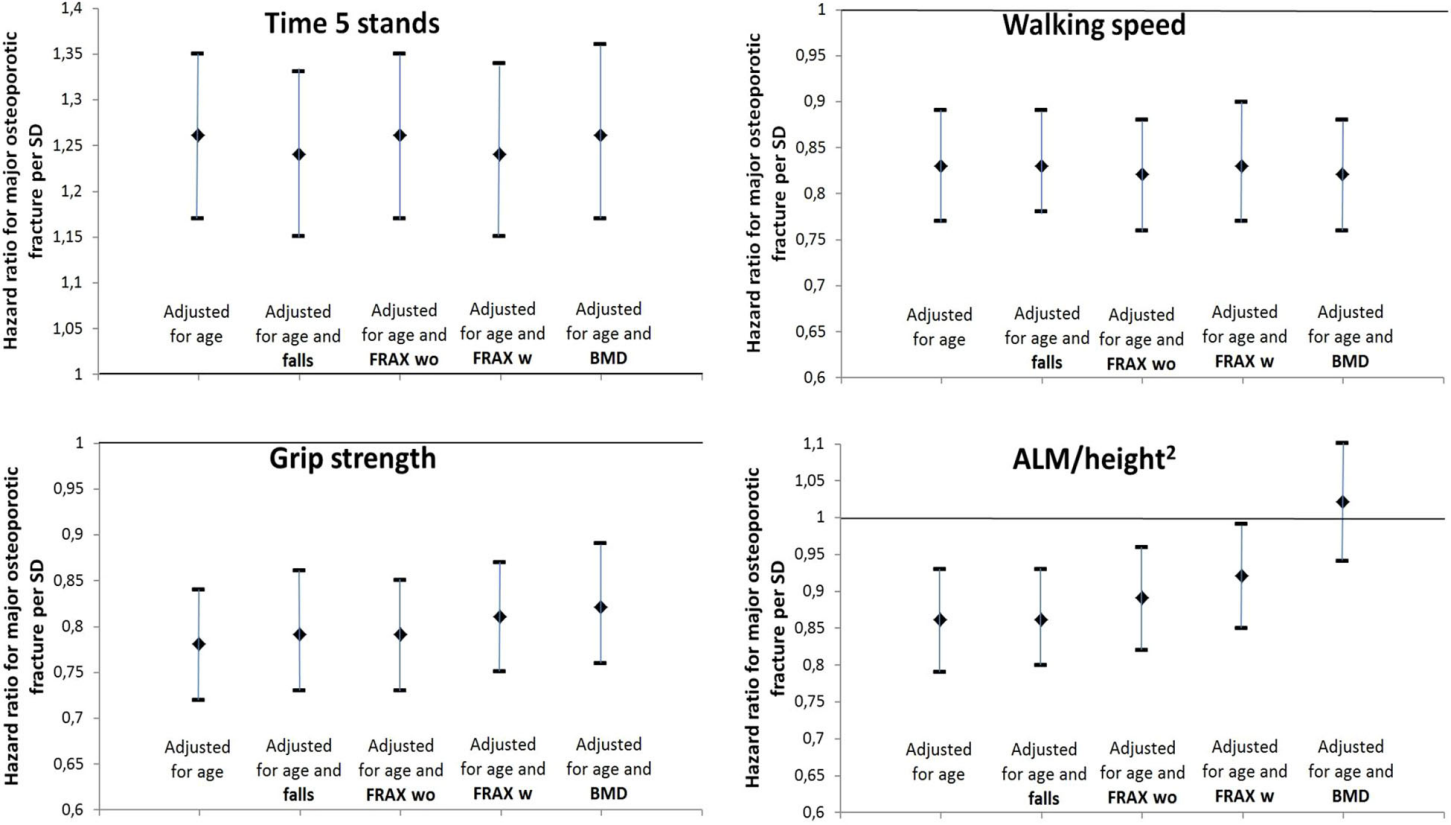
Associations between exposures and risk of incident fracture. Data are hazard ratio for fracture per 1 SD change in predictor (HR/SD), adjusted for age, follow‐up time, and as specified (in a subset of *N* = 7531 participants: *n* = 1661 Hong Kong; *n* = 1732 Sweden; *n* = 4138 United States).

Inclusion of femoral neck aBMD *T*‐score (Table 3) had a very modest attenuating effect on predictive value of chair stand time, walking speed, and grip strength, but completely removed associations between ALM/height^2^ and each of the nonhip fracture outcomes (HRs 1.01 to 1.02). Furthermore, the HR for hip fracture inverted to suggest a detrimental effect of increasing lean mass on hip fracture risk after adjustment for aBMD (HR 1.12; 95% CI, 1.01 to 1.23). Figure 1 depicts the effect of the different adjustments, using the participants in whom FRAX data were available.

### Interactions with age and follow‐up time

In models incorporating age or follow‐up time as interaction terms, there was no evidence that either variable influenced the predictive value of any of the four exposures. Thus, for chair stand time, the HR for any fracture was 1.08 (95% CI, 0.98 to 1.19) at age 70 years and 1.15 (95% CI, 1.08 to 1.21) at 80 years, *p* interaction = 0.12. The HR for any fracture with walking speed was 0.88 (95% CI, 0.81 to 0.95) at 1 year after baseline and 0.94 (95% CI, 0.87 to 1.01) at 10 years after baseline, *p* interaction = 0.28. All other interaction terms were *p* > 0.30.

## Discussion

We have demonstrated, in this large population cohort of older men, that physical performance (chair stand time, walking speed, grip strength) and ALM predict incident fracture risk independently of FRAX probability and history of prior falls. Though chair stand time, walking speed, and grip strength also predicted fracture risk independently of femoral neck aBMD (albeit with a slightly attenuated effect size), the inclusion of aBMD directly, or as part of FRAX, markedly attenuated associations between ALM and incident fracture.

There are several studies across a range of cohorts that demonstrate the predictive value of measures such as chair stand time, walking speed, and grip strength for fractures. The associations we have observed are consistent with those for physical performance, fractures, and falls derived using a different analytic methodology in the US MrOS cohort.^3^, ^4^, ^5^, ^28^, ^29^ In the present analysis, however, we have, across the three MrOS cohorts, extended such findings by demonstrating that the associations between these risk factors and incident fracture are independent of key clinical factors such as prior falls, BMI, and FRAX probability. Associations between appendicular lean mass and fracture reported in previous studies are mixed, with no association between ALM/height^2^ and hip fracture found in the US MrOS cohort^30^ or in women in the Framingham study,^31^ whereas a study of Swiss retirees found that low lean mass was a risk factor for clinical fractures.^32^

The attenuation (and indeed inversion for hip fracture) of the relationships between ALM and incident fracture by the inclusion of femoral neck aBMD are intriguing. A similar finding was observed in the Women's Health Initiative^33^ and in the Health ABC study,^34^ with the authors of the latter study suggesting that excess lean in excess of bone mass might be a profracture state. However, this would seem to be at odds with the general adaptation of bone to muscle,^35^ and excess lean mass or power over bone strength seems unlikely in older men (compared with younger athletes, for example). In contrast, in the Swiss GERICO (Geneva Retired Workers cohort) study, adjustment of low lean mass for aBMD did not substantially attenuate associations with incident fracture.^32^ Importantly, both the measure of lean mass and aBMD are derived from the same instrument, namely DXA, and were moderately correlated with a Pearson correlation coefficient ranging from 0.29 (USA) to 0.43 (Hong Kong). It is well established that soft tissue can influence the measurement of aBMD, potentially through a magnification artifact associated with a thicker body where BMI is higher, and through altered edge detection.^36^ This phenomenon has been particularly discussed in terms of adipose tissue; the effect of muscle mass, which is not specifically measured by DXA (it is derived as the tissue that is not fat or bone), has been much less thoroughly considered. Interestingly, the effect was very similar when ALM rather than ALM/height^2^ was used (data not shown), suggesting that it is not solely a result of size adjustment, although both ALM and ALM/height^2^ are strongly related to body size. The marked attenuation of associations using ALM/BMI is likely to be a consequence of ALM being a component of body weight (together with fat mass and bone mass), with BMI calculated as weight divided by height squared. Importantly, aBMD is calculated from equations incorporating soft tissue mass^36^; thus the possibility of measurement artifact must be considered. Assessment of muscle using an alternative modality, such as pQCT, might offer a potential route to clarification of this issue.

We studied three well‐characterized cohorts drawn from general populations with standardized assessments and prospective recording of fractures. However, there are some limitations that should be considered in the interpretation of our findings.^18^ First, the population studied was male, and of a narrow age range (64 to 99 years), thus limiting the generalizability of our findings. Second, the definition of glucocorticoid use differed from those usually specified for incorporation into FRAX. Third, there was no information on causes of secondary osteoporosis (other than rheumatoid arthritis and glucocorticoids), and this variable was therefore set to null. The effect of these considerations on our findings is uncertain, but may have led to an underestimation of risk by FRAX. Fourth, we were limited to DXA measures of lean mass, so that both lean and bone measures were obtained from the same scanner—DXA only approximates muscle mass. Finally, we did not specifically investigate any additional effect of multiple falls, and did not have information on the severity of a past fall, or whether a past fall was associated with injury, hence limiting our ability to identify events potentially most likely to be associated with a fracture outcome.

Although these results clearly demonstrate that measures such as chair stand time, walking speed, grip strength, and ALM offer risk information over and above FRAX with aBMD, how these might be incorporated into clinical assessment will require further investigation. An important consideration is whether the specific component of risk informed by each of these measures is amendable to intervention. Thus far, there are no medications licensed for the improvement of any of these measures, and there is no evidence for the efficacy of currently used antiosteoporosis therapies among individuals selected on the basis of such risk factors. Indeed, there is scant evidence that nonpharmacological interventions, for example, alterations to diet and/or physical activity to improve physical performance, actually reduce fracture risk.^37^, ^38^ For the moment then, these measures are most likely to be of adjunctive use in clinical decision making, perhaps to guide interventions for those close to intervention thresholds derived from FRAX and aBMD assessment, but also as the basis for directed nonpharmacological therapeutic approaches focused, for example, on reducing the risk of falls.^37^, ^38^ They may also be particularly relevant in older frail patients, who are often assessed in the context of multidisciplinary falls/ frailty clinics.

In conclusion, we have demonstrated that physical performance (chair stand time, walking speed, grip strength) and ALM are predictive of incident fractures, independently of prior falls and FRAX probability. The observation that inclusion of aBMD in the models markedly attenuated the predictive value of ALM requires further investigation to differentiate a true effect from artifact caused by DXA technology. Although our findings support the consideration of these measures in fracture risk assessment, further prospective studies in cohorts with wider age ranges, other ethnicities, and most importantly women, are now warranted to replicate and extend these findings, ideally to establish the potential for their inclusion as a modifier of FRAX probability.

## Disclosures

All authors have no disclosures in relation to this manuscript.

## Supporting information

Supporting Table S1.
