# Supplementary material for: Measures of Physical Performance and Muscle Strength as Predictors of Fracture Risk Independent of FRAX, Falls, and aBMD: A Meta‐Analysis of the Osteoporotic Fractures in Men (MrOS) Study
Source: J Bone Miner Res. 2018 Aug 29;33(12):2150–7. doi: 10.1002/jbmr.3556 (PMC6272117; doi:10.1002/jbmr.3556)
Supplement: Supplementary file 1 — Supporting Table S1. [file JBMR-33-2150-s001.docx]

**Measures of physical performance and muscle strength as predictors of fracture risk independent of FRAX, falls and BMD: A meta-analysis of the Osteoporotic Fractures in Men (MrOS) Study**

**Harvey NC et al.**

**Online supplementary material**

**Online Supplementary Table 1:** Associations between exposures and risk of incident fracture. Data are Hazard ratio for fracture (fx) per 1 SD change in predictor (HR/SD), adjusted for age, follow-up time, and additional adjustment for either: prior falls; FRAX MOF without femoral neck BMD; FRAX MOF with femoral neck BMD; femoral neck BMD. Statistically significant associations (p<0.05) are in bold.

| **Exposure (SD)** | **Adjustment** | **Any fx** | **Ost fx** | **MOF fx** | **Hip fx** |
| --- | --- | --- | --- | --- | --- |
| **Time 5 chair stands** | Age, FU time | **1.15 (1.10, 1.21)** | **1.19 (1.13, 1.25)** | **1.26 (1.19, 1.34)** | **1.36 (1.24, 1.49)** |
|  | + BMI | **1.17 (1.12, 1.22)** | **1.21 (1.15, 1.27)** | **1.28 (1.20, 1.36)** | **1.39 (1.27, 1.53)** |
|  | or + PA | **1.15 (1.09, 1.20)** | **1.17 (1.11, 1.24)** | **1.23 (1.16, 1.31)** | **1.32 (1.20, 1.45)** |
|  |  |  |  |  |  |
| **Walking speed** | Age, FU time | **0.91 (0.86, 0.95)** | **0.88 (0.83, 0.93)** | **0.85 (0.79, 0.90)** | **0.70 (0.64, 0.77)** |
|  | + BMI | **0.90 (0.85, 0.94)** | **0.87 (0.82, 0.91)** | **0.83 (0.78, 0.88)** | **0.69 (0.62, 0.76)** |
|  | or + PA | **0.91 (0.87, 0.96)** | **0.89 (0.84, 0.95)** | **0.86 (0.81, 0.91)** | **0.72 (0.66, 0.80)** |
|  |  |  |  |  |  |
| **Grip strength** | Age, FU time | **0.83 (0.79, 0.86)** | **0.79 (0.75, 0.83)** | **0.77 (0.72, 0.82)** | **0.72 (0.65, 0.79)** |
|  | + BMI | **0.83 (0.79, 0.87)** | **0.79 (0.75, 0.84)** | **0.77 (0.72, 0.82)** | **0.72 (0.65, 0.80)** |
|  | or + PA | **0.84 (0.80, 0.88)** | **0.80 (0.75, 0.84)** | **0.78 (0.74, 0.83)** | **0.74 (0.67, 0.82)** |
|  |  |  |  |  |  |
| **ALM/Height^2^** | Age, FU time | **0.89 (0.84, 0.93)** | **0.88 (0.83, 0.93)** | **0.85 (0.80, 0.90)** | **0.86 (0.78, 0.95)** |
|  | + BMI | **0.87 (0.81, 0.93)** | **0.87 (0.81, 0.93)** | **0.84 (0.77, 0.91)** | 0.90 (0.79, 1.02) |
|  | or +BMI and FN BMD | **0.92 (0.86, 0.98)** | 0.93 (0.87, 1.00) | **0.90 (0.83, 0.98)** | 1.01 (0.89, 1.14) |
|  | or + PA | **0.89 (0.85, 0.93)** | **0.88 (0.84, 0.93)** | **0.87 (0.81, 0.92)** | **0.87 (0.79, 0.96)** |
|  | or + PA and FN BMD | 1.01 (0.97, 1.06) | 1.03 (0.97, 1.08) | 1.04 (0.97, 1.11) | **1.14 (1.03, 1.26)** |
|  |  |  |  |  |  |
| **ALM/BMI** | Age, FU time | 1.00 (0.95, 1.04) | 0.99 (0.94, 1.05) | 0.99 (0.94, 1.05) | 1.05 (0.96, 1.15) |
|  | + falls | 1.00 (0.96, 1.04) | 0.99 (0.95, 1.05) | 0.99 (0.94, 1.05) | 1.05 (0.96, 1.16) |
|  | or + FRAX wo BMD | 0.97 (0.92, 1.03) | 0.98 (0.92, 1.04) | 0.99 (0.92, 1.06) | 1.05 (0.94, 1.17) |
|  | or + FRAX with BMD | 1.00 (0.94, 1.05) | 1.01 (0.95, 1.08) | 1.02 (0.95, 1.09) | 1.06 (0.95, 1.18) |
|  | or + FN BMD | 1.00 (0.95, 1.04) | 0.99 (0.94, 1.04) | 0.98 (0.93, 1.04) | 1.05 (0.96, 1.15) |

BMI = body mass index; PA = physical activity (PASE score); FN = femoral neck
